# A single-center, prospective, randomized clinical trial to investigate the optimal removal time of the urinary catheter after laparoscopic anterior resection of the rectum: study protocol for a randomized controlled trial

**DOI:** 10.1186/s13063-019-3210-1

**Published:** 2019-02-15

**Authors:** Lai Xu, Zhi-Yan Tao, Jun-Yang Lu, Guan-Nan Zhang, Hui-Zhong Qiu, Bin Wu, Guo-Le Lin, Tao Xu, Yi Xiao

**Affiliations:** 1 Department of General Surgery, Peking Union Medical College Hospital, Chinese Academy of Medical Sciences and Peking Union Medical College, No.1 Shuai Fu Yuan, Dongcheng District, Beijing, 100730 People’s Republic of China; 2 Department of Epidemiology and Statistics, Institute of Basic Medical Sciences, Chinese Academy of Medical Sciences/School of Basic Medicine, Peking Union Medical College, #5 Dongdan San Tiao, Beijing, 100005 People’s Republic of China

**Keywords:** Removal time, Urinary catheter, Acute urinary retention, Urinary tract infection, Laparoscopic anterior resection of the rectum

## Abstract

**Background:**

Urinary catheter placement is essential before laparoscopic anterior resection for rectal cancer. Whether early removal of the catheter increases the incidence of urinary retention and urinary tract infection (UTI) is not clear. This study aims to determine the optimal time for removal of the urinary catheter after laparoscopic anterior resection of the rectum.

**Methods/design:**

A total of 220 participants meeting the inclusion criteria will be randomly assigned to an experimental group or a control group. The experimental group will have their urethral catheters removed on postoperative day 2 and the control group will have their urethral catheters removed on postoperative day 7. In both groups, catheter removal will be performed when the bladder is full. The incidence of urinary retention and UTI in the two groups will be compared to determine the optimal catheter removal time.

**Discussion:**

This is a prospective, single-center, randomized controlled trial to determine whether early removal of the urinary catheter after laparoscopic anterior resection of the rectum will help to decrease the incidence of postoperative acute urinary retention and UTI.

**Trial registration:**

ClinicalTrials.gov, NCT03065855. Registered on 23 February 2017.

**Electronic supplementary material:**

The online version of this article (10.1186/s13063-019-3210-1) contains supplementary material, which is available to authorized users.

## Background

A urinary catheter is routinely placed before abdominal surgery to allow good visualization of the operative field. The catheter helps to avoid accidental trauma to the bladder and facilitates monitoring of kidney function during and after surgery. It is especially useful during anterior resection of the rectum because of the high risk of nerve injuries that could result in urinary retention or bladder dysfunction. The time for removal of the catheter varies in different operations. Traditionally, the catheter is retained for 7 days because early removal of the urinary catheter is reported to be associated with a higher incidence of urinary retention.

Generally, the longer the catheter is retained, the higher the risk of urinary tract infection (UTI). The recent Enhanced Recovery After Surgery (ERAS) guideline, taking into account the discomfort and anxiety suffered by patients, recommends that urinary catheters placed via the urethra be withdrawn 48 h after colon/rectal surgery in patients receiving epidural pain relief [1]. However, the literature referenced in the guideline mainly included patients undergoing colorectal surgery and not anterior resection of the rectum, which is the focus of our research. Most authors still believe that a longer time of indwelling catheterization can reduce the possibility of acute urinary retention and long-term bladder dysfunction.

During our review of the literature we found only one randomized controlled trial (published in 1999) indicating that early removal of the catheter was associated with higher risk of urinary retention [2]. Recent studies have reported contradictory results, with some authors claiming that early removal of the urinary catheter increases the risk of urinary retention [3] and others claiming the opposite [4]. Advances in laparoscopic techniques have made it possible to clearly visualize and preserve the superior hypogastric nerve and the pelvic nerve (which are associated with urinary function) during radical surgery. In addition, with the introduction of dissection along the embryonic plane and the use of neoadjuvant therapy for tumor reduction, protection of the nerves has become easier.

The purpose of this prospective, randomized controlled study is to compare the incidences of acute urinary retention and UTI in patients having catheter removal at 2 days and 7 days after laparoscopic anterior resection and thus establish the optimal time for catheter removal. The primary outcome measure is the incidence of urinary retention. The secondary outcome measures are the incidence of UTI, time of off-bed activity, and subjective discomfort. The study protocol has been written in accordance with the Standard Protocol Items: Recommendations for Interventional Trials (SPIRIT) checklist (Additional file 1).

## Methods

### Study design

**Fig. 1:**
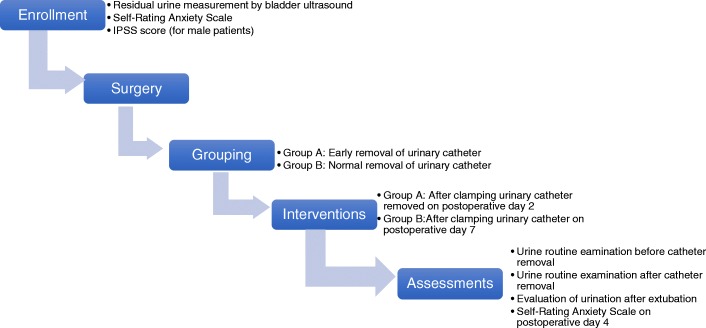
Flow diagram of enrollment, intervention, and assessment. IPSS International Prostate Symptom Score

This study is a superiority trial and is designed as a prospective, single-center, randomized, parallel-group, single-blind, clinical controlled study. Rectal cancer patients will be enrolled and divided into two groups: the early removal group (the intervention group) and the normal removal group (the control group). The flow diagram for this trial is shown in Fig. 1.

The sample size was estimated as follows. According to the latest studies, the incidence of urinary retention after rectal surgery is 25% when the catheter is removed within 2 days after surgery and 10% when the catheter is removed after 7 days. To detect these outcomes with *α* = 0.05 and *β* = 0.2, we would need 100 patients per group (total 200). We decided to enroll 110 patients in each group (total 220), to allow for a possible 10% dropout rate.

### Outcome measures

#### Primary outcome measures

1. Postoperative urinary retention requiring recatheterization following urethral catheter removal.

Acute postoperative urinary retention is defined as > 100 ml postvoid residual urine demonstrated on bladder ultrasound, with need for recatheterization within 1 day of removal of the urethral catheter [2].

#### Secondary outcome measures

1. Urinary tract infection occurring following urethral catheter removal.

A midstream urine sample will be taken for microscopy and culture before catheter removal. A urinary leukocyte count ≥ 5 per high-power field for men and ≥ 1 per high-power field for women will be considered as bacteriuria, irrespective of whether or not symptoms of urinary irritation are present. A pure culture of a single organism of > 100,000 colony forming units will be considered a positive culture [2].

(2) Urethrorrhagia following urethral catheter removal.

The presence of ≥ 3 red blood cells (RBC) per high-power field will be recorded as urethrorrhagia [2].

(3) Anxiety score before operation and on postoperative day 4.

The Zung Self-Rating Anxiety Scale will be used to assess anxiety levels. This is a standard scale for measurement of anxiety, and is mainly used to assess the efficacy of treatments [5].

### Participating centers and investigators

This will be a single-center study, with all patients enrolled from the Peking Union Medical College Hospital.

The surgeons participating in this study will have performed > 100 laparoscopic colorectal surgeries per year for 2 consecutive years and will have proven ability to complete the laparoscopic surgery procedure. Every investigator will participate in training on the correct technique for removal of urinary catheters.

### Ethical considerations

This trial protocol has been approved by the Ethics Committee of Peking Union Medical College Hospital (ZS-1269) and has been registered at Clinical-Trials.gov (identifier NCT03065855). All eligible participants and/or their legal surrogates will be fully informed of the potential risks and benefits of the interventions in each group. Only patients who provide written informed consent will be enrolled in the trial.

### Study subjects

The study participants will be rectal cancer patients requiring laparoscopic anterior resection of the rectum.

#### Inclusion criteria

- Age 18–75 years.
- Diagnosed with rectal cancer and posted for total or tumor-specific mesorectal excision with colorectal or coloanal anastomosis.
- American Society of Anesthesiologists (ASA) classification of 1–3.

#### Exclusion criteria

- Preoperatively diagnosed urinary tract infections or urinary system diseases (including end-stage renal disease, neurological bladder dysfunction, and malignancy).
- Previous history of urinary retention or of having received drugs likely to affect bladder function.
- Male patients with disease of the prostate (such as benign prostatic hyperplasia).
- Patients receiving emergency surgery.

#### Exit criteria

- Rectal cancer surgery is combined with additional pelvic surgery (including pelvic lymph node dissection, hysterectomy, salpingo-oophorectomy, posterior vaginectomy, cystectomy, ureteral double-J stenting, ureterectomy, ureteroureterostomy, prostatectomy).
- There is significant intraoperative damage to the pelvic nerve (patients classified as type IV by the Sugihara standard).
- Postoperative Clavien–Dindo grade III or VI complications.

### Randomization and blinding

Patients will be randomized to the study group and the control group with an allocation ratio of 1:1. Randomization will be done using random numbers generated with Statistical Analysis System (SAS) software. A central randomization system will be employed. All of the researchers will register on the website of the research system. When a researcher has chosen a certain patient, he/she will log in to the webpage and upload the basic information on the patient. The group allotment will then be revealed.

All patients will be undergoing the same (similar) surgery. The only difference will be the catheter removal time. Obviously, it is impossible to blind the surgeons who will perform the operations. However, all surgeon researchers will be required to limit potential cointerventions as much as possible. Result assessment will be made by independent researchers blinded to the surgical procedures.

### Preoperative evaluation

Preoperative evaluation will include the following:

- Elicitation of history of previous urinary system disease.
- Evaluation of bladder function and residual urine by B-mode ultrasonography.
- Urine routine examination to confirm that there is no pre-existing urinary tract infection or hematuria.

### Grouping

All participants will undergo urethral catheter insertion after placement of the epidural catheter for analgesia. After urethral catheter placement the participants will be randomly assigned to either the experimental arm or the control arm.

Participants assigned to the experimental arm will have their urethral catheters removed on postoperative day 2, and participants assigned to the control group will have their urethral catheters removed on postoperative day 7. In both groups, catheter removal will be done when the bladder is distended; this is standard practice in our institution. Control group patients will practice intermittent catheter clamping from postoperative day 4 onward.

### Detailed description of procedure

Catheterization will be performed using all aseptic precautions. The investigator will ensure that the drainage bag is positioned intraoperatively and postoperatively. The drainage tube ends and drainage bags will not be higher than the pubic symphysis, and will be kept unobstructed. The urethra, urethral orifice, and perineum will be kept clean when removing the catheter so that a clean midstream urine sample can be collected for urine routine examination and culture. The catheter retention duration will be recorded, and also the first self-urination time, residual urine, pain score, and recatheterization (if any).

For all patients the catheter will be clamped for some time so that the bladder is full when the catheter is removed. The catheter will be pulled out when the desire for urination is at a peak and bladder pressure is increasing. These measures are conducive to the recovery of bladder function and establishment of an effective micturition reflex, which can effectively shorten the time to first micturition after catheter removal.

### Statistical analysis

Continuous variables will be summarized as means (with standard deviation), medians (with Q1–Q3 or interquartile range), and minimum and maximum values. Categorical variables will be expressed as numbers and percentages. The *t* test and Wilcoxon rank-sum test will be used to compare continuous variables, and the chi-square test or Fisher’s exact test to compare categorical variables. Survival analysis will be conducted using the Kaplan–Meier method and the log-rank test. Statistical analysis will be carried out using SAS 9.2. The intention-to-treat principle will be followed during analysis, but patients who develop serious complications in the perioperative period will be excluded. All statistical tests will be two-sided, and *P* ≤ 0.05 will be considered statistically significant.

## Discussion

Most researchers still believe that longer retention of the urinary catheter after surgery reduces the incidence of acute urinary retention and bladder dysfunction [6]. Early catheter removal and resulting urinary retention leads to recatheterization and, thus, to higher risk of UTI. However, overlong retention of the catheter has been shown to cause permanent injury to the detrusor in animal experiments [7].

Urinary retention refers to an inability to completely empty the bladder. Despite a large amount of collected urine, the patient will not have the desire to micturate. Several factors contribute to the occurrence of urinary retention, including surgical trauma, the procedure of catheter removal, and prolonged catheterization.

Damage to nerve fibers from S3–S4 spinal segments during rectal surgery could lead to detrusor dysfunction and urinary retention [8]. This is most commonly reported after low anterior resection (LAR; 15–25%), abdominoperineal resection (APR; ~ 50%), and laparoscopic surgery [9]. Deeper anesthesia and longer duration of surgery are also associated with a delay in recovery of the micturition reflex [10]. Anxiety also leads to a delay in the recovery of bladder function.

The procedure of catheter removal is very important. Clinical trials show that the catheter is ideally removed when the bladder is distended. The increased bladder pressure helps the detrusor contract strongly after catheter removal, and this facilitates recovery of the micturition reflex. Therefore, in this study all investigators will undergo training to standardize the procedure of catheter removal. Prolonged catheterization leads to loss of bladder smooth muscle tone, which may also lead to bladder sphincter dysfunction [11]. In addition, prolonged catheter retention irritates the urethral mucosa and causes inflammatory reaction and edema. All of these factors contribute to the occurrence of urinary retention.

There have been few randomized controlled trials to investigate the optimal catheter removal time after rectal cancer surgery. In a randomized clinical trial involving 126 patients, Benoist et al. [2] found that the acute urinary retention rate was significantly higher when catheter removal was on postoperative day 1 than when it was on postoperative day 5 (25% vs. 10%; *P* < 0.05), but the incidence of UTI was significantly lower (20% vs. 42%; *P* < 0.01). However, it must be noted that protection of the pelvic nerves is much better in current laparoscopic surgery. The sample size of the cited study was also relatively small. Yoo et al. [4] retrospectively analyzed the data of 224 patients undergoing LAR, APR, and total colorectal resection with ileal pouch–anal anastomosis (IPAA). They found no significant difference in urinary retention rates between patients receiving catheter removal on postoperative day 1 vs. patients receiving catheter removal any time after postoperative day 1 (4.8% vs. 4.7%; *P* = 1.0). Another study has shown the opposite; that is, early removal of the ureteral catheter is an independent risk factor for acute urinary retention [3]. The incidence of acute urinary retention in the early removal group (within 2 days) was 30.8%, which was significantly higher than the rate in the sample as a whole (20%); however, the incidence of UTI in the early removal group was not significantly higher (15.4% vs. 8.9%; *P* = 0.29).

To summarize, there is a paucity of high-level evidence regarding the optimal time for urinary catheter removal after rectal surgery, and so far there is no consensus among different research groups. Determining the optimal urinary catheter removal time for patients undergoing laparoscopic anterior resection of the rectum will be useful for reducing patient discomfort and for early patient mobilization after surgery. Therefore, the results of the proposed clinical trial should be of great help to surgeons.

## Trial status

This trial was initiated in July 2017 and is currently recruiting patients.

## Additional file

Additional file 1: SPIRIT 2013 Checklist: recommended items to address in a clinical trial protocol and related documents. SPIRIT Figure: recommended content for the schedule of enrolment, interventions, and assessments. (DOCX 48 kb)

## Abbreviations

APR: Abdominoperineal resection
ASA: American Society of Anesthesiologists
ERAS: Enhanced Recovery After Surgery
IPAA: Ileal pouch–anal anastomosis
LAR: Low anterior resection
SAS: Statistical Analysis System
UTI: Urinary tract infection
